# Multi-omics analysis reveals molecular mechanisms of shoot adaption to salt stress in Tibetan wild barley

**DOI:** 10.1186/s12864-016-3242-9

**Published:** 2016-11-07

**Authors:** Qiufang Shen, Liangbo Fu, Fei Dai, Lixi Jiang, Guoping Zhang, Dezhi Wu

**Affiliations:** Department of Agronomy, Key Laboratory of Crop Germplasm Resource of Zhejiang Province, Zhejiang University, Hangzhou, 310058 China

**Keywords:** Barley (*Hordeum vulgare*), Ionome, Metabolome, Proteome, Salinity

## Abstract

**Background:**

Tibetan wild barley (*Hordeum spontaneum L.*) has been confirmed to contain elite accessions in tolerance to abiotic stresses, including salinity. However, molecular mechanisms underlying genotypic difference of salt tolerance in wild barley are unknown.

**Results:**

In this study, two Tibetan wild barley accessions (XZ26 and XZ169), differing greatly in salt tolerance, were used to determine changes of ionomic, metabolomic and proteomic profiles in the shoots exposed to salt stress at seedling stage. Compared with XZ169, XZ26 showed better shoot growth and less Na accumulation after 7 days treatments. Salt stress caused significant reduction in concentrations of sucrose and metabolites involved in glycolysis pathway in XZ169, and elevated level of tricarboxylic acid (TCA) cycle, as reflected by up-accumulation of citric acid, aconitic acid and succinic acid, especially under high salinity, but not in XZ26. Correspondingly, proteomic analysis further proved the findings from the metabolomic study.

**Conclusion:**

XZ26 maintained a lower Na concentration in the shoots and developed superior shoot adaptive strategies to salt stress. The current result provides possible utilization of Tibetan wild barley in developing barley cultivars for salt tolerance.

**Electronic supplementary material:**

The online version of this article (doi:10.1186/s12864-016-3242-9) contains supplementary material, which is available to authorized users.

## Background

Soil salinity is one of major abiotic stresses for plants in the world, posing a great threat to agricultural production. At present, approximately 20 % of the globally cultivated land and nearly half of the total irrigated land are adversely affected by salinity [1, 2]. On the other hand, salt tolerance shows a wide variation in plant species. Among cereals, rice (*Oryza sativa*) is the most sensitive, and durum wheat (*Triticum turgidum*) is sensitive and bread wheat (*Triticum aestivum*) is moderately tolerant; while barley (*Hordeum vulgare*) is the most tolerant [3]. Consequently, barley is often considered as an excellent model crop in the attempts to understand the mechanisms of salt tolerance in cereal crops.

Barley is the fourth most important cereal crop in the world in terms of the planting area, only behind maize (*Zea mays*), wheat and rice, and it is mainly used as animal feed and raw material in brewing industries [4]. Moreover, barley is well-known for its wide adaptability to various environments [4, 5]. However, the genetic diversity of the cultivated barley becomes narrower due to artificial activities, including modern breeding and intensive planting. Comparatively, wild barley accessions (*H. vulgare ssp. spontaneum*) and other *Hordeum* species contain wider genetic diversity and are rich in elite alleles [6, 7]. It was reported that the cultivated barley contained only around 40 % of alleles in *H. spontaneum* [6]. Obviously, wild barley may provide novel alleles or genes for breeding, in particular those with high tolerance to abiotic stresses [7].

Wild barley is the progenitor of cultivated barley and mainly distributed in Mediterranean area, Fertile Crescent and Qinghai-Tibet plateau [5, 8]. A large number of wild barley accessions collected from Qinghai-Tibet region (referred to Tibetan wild barley thereafter) display wide genetic diversity and closely genetic homology to cultivated barley, and are recently proved equal contribution to the genome of modern cultivated barley as the wild barley from the Near East Fertile Crescent [8]. Many accessions with strong tolerance to abiotic stresses, including drought, salinity and aluminum toxicity, were identified from Tibetan wild barley [9–11]. For example, in our previous study, we identified elite salt-tolerant genotypes from around 200 accessions of Tibetan wild barley (e.g. XZ16 and XZ26), showing a better salinity tolerance than CM72, a well-known salt-tolerant cultivar [12].

Evaluation of salt tolerance among different species or genotypes within a species is quite difficult by one trait [13]. So far, many physiological parameters were used to identify salt tolerance, including relative root length [14], leaf cell elongation [15], relative dry weights of shoots and roots [10, 12], Na and K contents [16, 17], and K/Na ratio [18, 19]. Among these traits, relative shoot dry weight might be a more real and reliable trait for reflecting plant growth under salt stress, as shoot growth is generally more sensitive than root growth in response to salinity. Munns and Tester [3] divided the inhibition of shoot growth into two phases: a rapid response to osmotic pressure and a slow response to toxic level of Na accumulation in plant tissues. A significantly negative correlation between shoot Na concentration and relative shoot dry biomass was observed among Tibetan wild barley accessions [10, 12]. Shoot growth is the fundamental for normal ontogenesis of plants and yield formation. Thus, it is imperative to reveal the mechanism of salt tolerance underlying shoot growth in order to developing the salt tolerant cultivars.

Many methods have been used to identify a single gene or multi-genes network responding to salt stress, including linkage (or QTL mapping) mapping, association mapping (GWAS), and high-throughput omic techniques, such as transcriptomics, ionomics, proteomics and metabolomics analysis [20]. In barley, many QTLs associated with salt tolerance have been identified [21, 22]. A single locus controlling salt tolerance, *HvNax3*, was identified on the short arm of chromosome 7H from a wild barley accession CPI-71284-48, which controls sodium (Na) accumulation in shoots under salt stress [23]. However, very few genes associated with salt tolerance have been cloned from these QTLs in barley, mainly because of its huge genome. Fortunately, omic methods are high-throughput and efficient for comprehensive understanding of salt-induced changes of gene-protein-metabolite system at genome-wide scale [20]. Previously, salt-induced changes of transcriptome, proteome, ionome and metabolome were revealed by comparing the salt tolerant cultivars with sensitive ones, or comparing the wild barley accessions with cultivars [24–27]. Compared with these findings at transcript level, the molecular responses at protein and metabolic levels are more close to the adaptive or tolerant mechanism under salt stress. These researches may provide some valuable information about the difference between salt-tolerant and sensitive genotypes in their response to salt stress; however, most of the experimental materials used in these researches did not show predominant difference in salt tolerance.

As mentioned above, we identified some accessions of Tibetan wild barley differing dramatically in salt tolerance (e.g. XZ26, tolerant; XZ169, sensitive) in our previous study [12]. However, the molecular mechanisms underlying the genotypic difference in salt tolerance are still unknown. Hence, XZ26 and XZ169 were used to investigate the changes of shoot ionome, metabolome and proteome in the response to salt stress of 200 and 400 mM at seedling stage, so as to understand the adaptive approach of barley shoots to salt stress.

## Results

### The influence of salt stress on shoot growth of the two wild barley genotypes

**Fig. 1:**
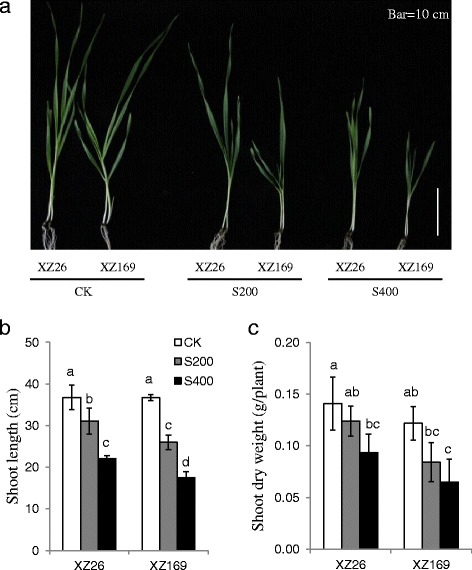
Shoot growth performance of XZ26 and XZ169 under control (CK), moderate (200 mM, S200) and high (400 mM, S400) salinity conditions. (**a**) Pictures of shoot-plants of XZ26 and XZ169 after 7 days salt treatment and control conditions, *bar* shows 10 cm; (**b**) Shoot length (cm) and (**c**) shoot dry weight (g/plant) of XZ26 and XZ169. Data are means ± SD of three biological replicates (*n* = 3) and different *small letters* indicate significant difference at *p* < 0.05 by the One-Way ANOVA test

Two wild barley genotypes, XZ26 and XZ169, which were identified from about 200 Tibetan wild barley accessions, differed greatly in salt stress tolerance [12]. Currently, shoot growth of the two genotypes was compared under moderate (200 mM) and high (400 mM) salinity (Fig. 1 and Additional file 1: Figure S1). After 7 days salt treatment, shoot length and biomass were significantly reduced for the two genotypes in comparison with their controls, with XZ26 reducing by 15.4 and 40.0 %, and XZ169 by 29.2 and 52.3 % in the shoot length under moderate and high salinity, respectively (Fig. 1b). Correspondingly, for the shoot dry weight, XZ26 reduced by 22.0 and 33.5 %, and XZ169 by 31.1 and 46.7 % under the two salt levels, respectively (Fig. 1c). Obviously, XZ26 showed the higher salt tolerance than XZ169 in terms of the shoot growth.

### The influence of salt stress on shoot ionome of the two wild barley genotypes

**Fig. 2:**
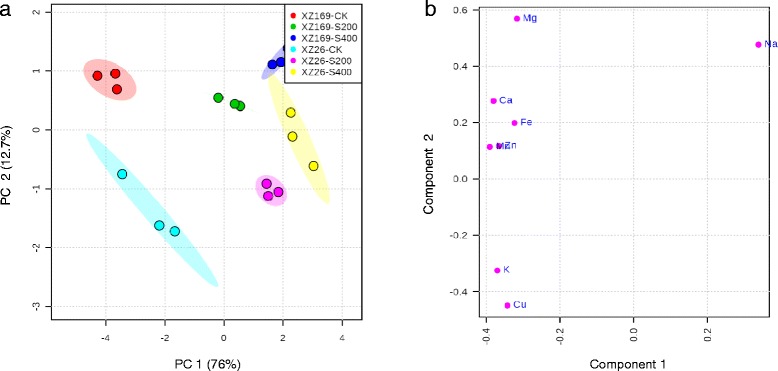
Shoot ionome variation in XZ26 and XZ169 and components of elements to the PC1 and the PC2. (**a**) Shoot ionome variation among samples detects by the PCA after 7 days salt treatment and control conditions; (**b**) the components of elements to the PC1 and the PC2. CK: controls; S200: 200 mM NaCl; S400: 400 mM NaCl (*n* = 3); PC1: the first principal component; PC2: the second principal component

**Fig. 3:**
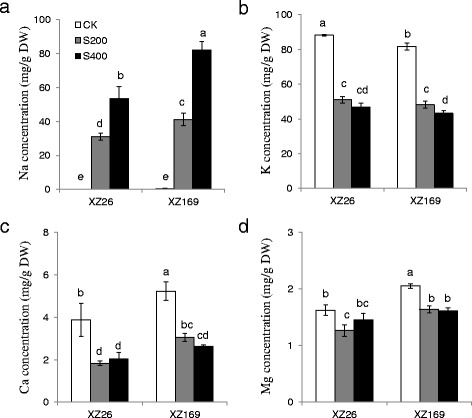
The concentration of Na, K, Ca and Mg in the shoots of XZ26 and XZ169 under control (CK), moderate (200 mM, S200) and high (400 mM, S400) salinity conditions. (**a**) Shoot Na concentration; (**b**) Shoot K concentration; (**c**) Shoot Ca concentration; (**d**) Shoot Mg concentration. Element concentration was determined in shoots of XZ26 and XZ169 after 7 days salt treatment and control conditions. Data are means ± SD of three biological replicates (*n* = 3) and different *small letter* indicates significant difference at *p* < 0.05 by One-Way ANOVA test

The control and treatment samples of both genotypes could be separated by the first principal component (PC1) using principal component analysis (PCA), which accounted for 76.0 % of the total variation in shoot ionomes (Fig. 2a). The most important factor contributing to the PC1 was Na (Fig. 2b). Shoot Na concentration was increased markedly when plants were exposed to salt treatments. Under 400 mM NaCl, Na concentrations in the two genotypes was almost increased by two folds in comparison with the plants under 200 mM NaCl (Fig. 3a).

Correspondingly, the PC2 could basically separate the samples between genotypes (Fig. 2a), and the major factors contributing to the PC2 were Na and Mg (Fig. 2b). XZ169 had distinctly higher shoot Na concentration than XZ26, being 1.3 and 1.5 fold under moderate and high salt levels, respectively (Fig. 3a). Meanwhile, the concentrations of other macroelements (K, Ca and Mg) and microelements (Cu, Fe, Mn and Zn) in shoots were significantly decreased for both genotypes under salt stress (Fig. 3b-d and Additional file 2: Figure S2). The changed pattern of these elements in shoots was quite similar for both genotypes, but XZ169 had higher shoot Mg concentration than XZ26, irrespectively of salt level. Obviously, the results indicate that XZ26 is a salt tolerant genotype with low shoot Na accumulation.

### The influence of salt stress on shoot metabolome of the two wild barley genotypes

**Fig. 4:**
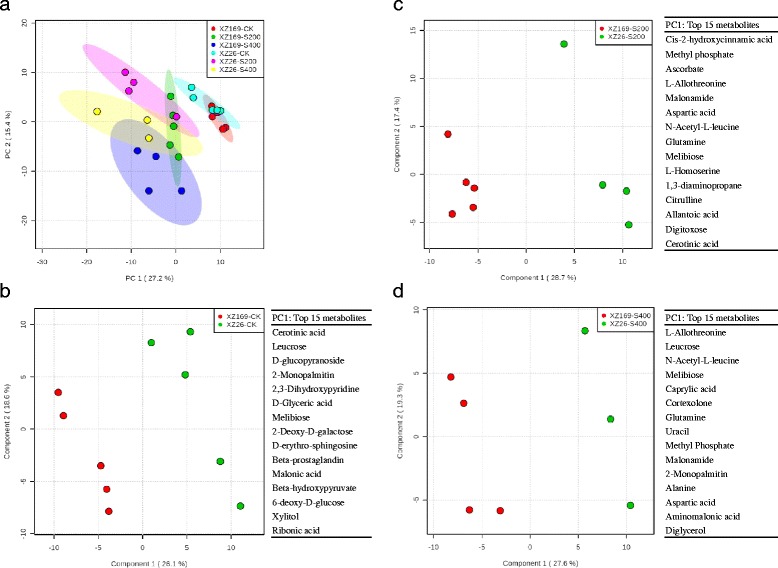
Shoot metabolome variation in XZ26 and XZ169 and top 15 metabolites for the PC1. (**a**) Shoot metabolome variation among samples detected by the PCA; (**b**-**d**) shoot metabolome variation among samples and the 15 top metabolites contributing to the PC1, were detected by the partial least squares-discriminant analysis (PLS-DA), respectively. Five biological replicates (*n* = 5) set for each treatment and samples with false determination were deducted from the data. CK: controls; S200: 200 mM NaCl; S400: 400 mM NaCl; PC1: the first principal component; PC2: the second principal component

Totally, 218 metabolites were identified in the shoots of the two genotypes (Additional file 3: Table S1). Shoot metabolomes of the two genotypes were dramatically changed under salt stress in comparison with their controls. The control and salt-treated samples could be clearly separated by the PC1, and the samples between the two genotypes were not well separated by the PC2 (Fig. 4a). Thus, the partial least squares-discriminant analysis (PLS-DA) was used to determine the difference between the two genotypes (Fig. 4b-d).

Without salt stress, the metabolites contributing to the PC1 were dominated by cerotinic acid, leucrose and other 13 metabolites between the two genotypes (Fig. 4b). Under moderate salt stress, the PC1 was dominated by hydroxycinnamic acid, methyl-phosphate, ascorbate and other metabolites (Fig. 4c). While under high salt stress, the dominated compounds were allothreonine, leucrose, N-acetyl-L-leucine and other 12 metabolites (Fig. 4d). Among them, 6 of the 15 top metabolites contributing to the PC1 were identified in the two salt treatments, but not in the controls, including allothreonine, N-acetyl-L-leucine, glutamine, methyl-phosphate, malonamide and aspartate.

**Fig. 5:**
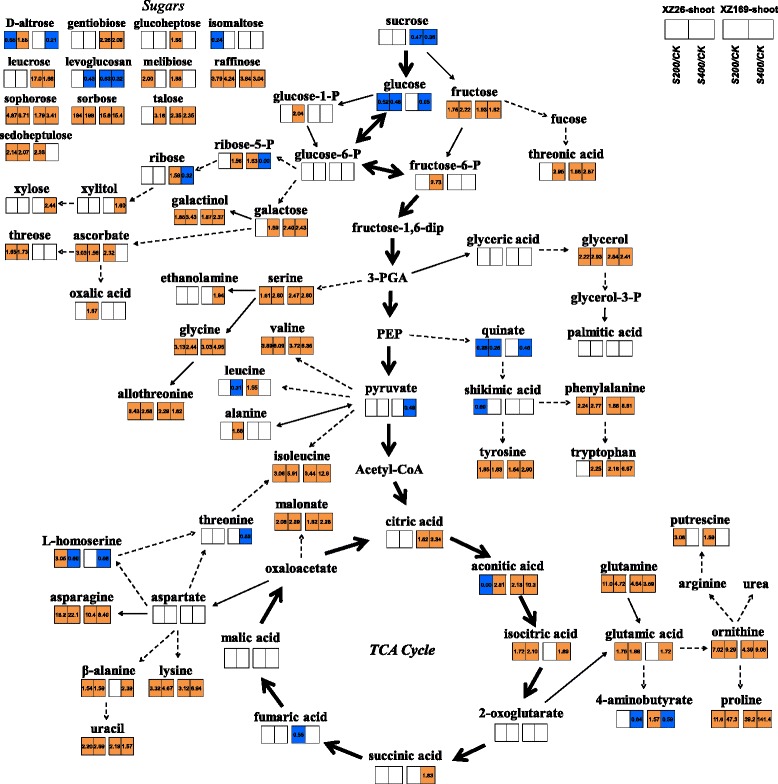
Changes in metabolites mapped to the metabolic pathways in the shoots of XZ26 and XZ169 after salt treatments. Metabolites in *orange* indicate significant (*P* < 0.05) up-accumulation and in *blue* show significant (*P* < 0.05) down-accumulation, in comparison of metabolite normalized content under salt stress and control conditions. Metabolic pathways were constructed according to KEGG (http://www.genome.jp/kegg/) metabolic database. CK: controls; S200: 200 mM NaCl; S400: 400 mM NaCl

Accumulation of some sugars, including fructose, raffinose, sophorose and sorbose, was increased in both genotypes under moderate and high salt level relative to control. Melibiose and sedoheptulose were up-accumulated specifically under moderate salinity, while under high salinity galactose and talose were dramatically increased in the two genotypes (Fig. 5). Some metabolites responded to salt stress in the genotype-dependent pattern. In details, salt stress caused a significant reduction of sucrose concentration (0.47- and 0.36 fold under moderate and high salt levels compared with the control, respectively) in XZ169, but the difference is not obvious in XZ26. The metabolites involved in glycolysis pathway also showed much larger down-accumulation in XZ169 than in XZ26 under high salinity, including glucose (0.05 fold) and pyruvate (0.49 fold); whereas glucose-1-P (2.04 fold) and fructose-6-P (2.73 fold) were up-accumulated in XZ26 and less changed in XZ169. Obviously, metabolic level of glycolysis was less affected by salt stress in the salt-tolerant XZ26 than in the sensitive genotype, XZ169 (Fig. 5).

The TCA cycle was significantly enhanced in XZ169 under salt stress, as reflected by up-accumulation of citric acid (3.34 fold), aconitic acid (10.3 fold), isocitric acid (1.89 fold) and succinic acid (1.83 fold), while no significant change was detected for citric acid and succinic acid in XZ26. The enhancement of the TCA cycle resulted in the dramatic increase of organic acids (e.g. aconitic acid, glycolic acid, lauric acid, picolinic acid, malonic acid and threonic acid) and amino acids (e.g. asparagines, β-alanine, glutamine, isoleucine, lysine, ornithine and proline) concentrations (Fig. 5 and Additional file 3: Table S1). For an instance, 2-oxoglutarate showed no significant change probably due to the enhanced synthesis of proline, a down-stream metabolite, which increased by 39.2 and 141.4 folds in XZ169 and by 11.6 and 47.3 folds in XZ26 under moderate and high salt levels, respectively (Fig. 5 and Additional file 3: Table S1).

In addition, allothreonine, N-acetyl-L-leucine, glutamine, methyl-phosphate, malonamide and aspartate, contributing to the separation of shoot metabolome between the two genotypes under salt stress, as mentioned above, had much higher concentration in XZ26 than in XZ169 (Fig. 5 and Additional file 3: Table S1).

### The influence of salt stress on shoot proteome of the two wild barley genotypes

**Fig. 6:**
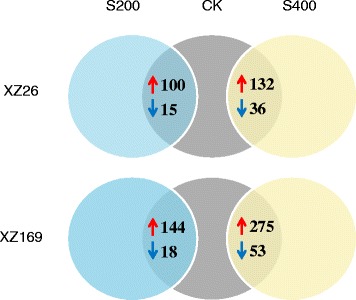
Global comparison of proteome profiles in the shoots of XZ26 and XZ169 after salt treatments. Totally 3358 proteins were identified by iTRAQ in shoots of XZ26 and XZ169 after 7 days salt treatment and control conditions and the *numbers* in the figure indicate the numbers of proteins with significant up-regulation (>1.5 fold) or down-regulation (<0.67 fold) for each comparison. *Red* and *blue arrows* represent up- and down- regulated proteins, respectively. CK: controls; S200: 200 mM NaCl; S400: 400 mM NaCl

Among 3358 proteins identified in the shoots, 100 and 15 proteins for XZ26, 144 and 18 proteins for XZ169, showed significant up-regulation and down-regulation under 200 mM NaCl compared with their controls, respectively (Fig. 6). Correspondingly, there were 132 and 36 proteins for XZ26, 275 and 53 proteins for XZ169, showing up-regulation and down-regulation under 400 mM NaCl, respectively (Fig. 6). The results indicate that shoot proteome is more dramatically changed under high salinity than under moderate salinity, and in the sensitive genotype than in the tolerant genotype. Gene ontology (GO) annotation of these differentially accumulated proteins showed that proteins involved in three major categories were most enriched after salt treatments including biological process, molecular function and cellular component, respectively (Additional file 4: Figure S3).

Among 442 differentially accumulated proteins, some showed genotype-dependent response to salt stress (Additional file 5: Table S2). In details, aquaporin (Q08IH4), apolipoprotein (F2D712), AAA-type ATPase (M0XZ33), vacuolar ATP synthase subunit (M0V7E0), reticulon-like protein (F2DHE3) and a conserved hypothetical protein (F2DTQ3), considered as membrane proteins, were significantly up-regulated in XZ169 under high salinity, but not in XZ26 (Table 1). However, some ion transporters, including potassium transporter (M0UZZ1), MRP (M0X1H9)- and PDR (M0WIH0)- like ABC transporter, nitrate/chlorate transporter (M0WRF7) and chloride channel 1 (E9LFE6) were not significantly regulated in the two genotypes under salt stress. Two proton pumps of plasma membrane H^+^-ATPase (F2DC32 and M0Z2H5) also showed no significant regulation under salt stress in both genotypes (Table 1). In fact, as mentioned above, the response of those transporters to salt stress could not explain the genotypic difference in shoot Na accumulation.

**Table 1:** The fold changes of proteins related to transporters in shoots of XZ26 and XZ169 under control (CK), 200 (S200) and 400 (S400) mM salt conditions

Uniprot ID	Protein description	MW (Da)	pI	AASC (%)	Score	XZ26		XZ169		XZ26/XZ169		
						S200/CK	S400/CK	S200/CK	S400/CK	CK	S200	S400
M0UXN8	Sugar transporter	59,996	8.26	8.1	90	0.93	1.51	0.67	0.57	0.54	0.75	1.44
M0W6D8	Sucrose transporter	36,282	8.57	4.7	41	0.90	1.11	0.95	0.87	1.01	0.95	1.30
M0V1P8	Cation/H^+^ exchanger domain containing protein	65,063	5.2	4.1	34	0.94	1.00	1.13	0.90	0.90	0.75	1.00
M0WKI5	Anion-transporting ATPase family protein	54,214	8.78	9.4	107	0.98	1.03	0.96	0.91	0.79	0.80	0.89
M0YDJ4	Phosphate transporter family protein	64,247	9.54	5.7	101	1.06	1.06	1.30	0.91	0.94	0.77	1.10
B0I531	Plasma membrane intrinsic protein	33,397	9.36	27.1	351	0.83	0.87	1.00	0.94	1.00	0.82	0.92
M0Z7X0	K/Mg/Cd/Cu/Zn/Na/Ca/Na/H-transporter domain containing protein (P-type)	10,009	5	21.9	81	0.94	0.97	0.93	0.94	1.03	1.04	1.06
F2CQF9	HvPIP2;1 protein	33,934	7.68	35.2	208	1.01	0.82	1.01	0.94	1.29	1.30	1.12
M0X1H9	MRP-like ABC transporter	122,841	6.41	11.4	171	0.94	0.92	1.10	0.97	1.02	0.88	0.97
F2DC32	Plasma membrane H^+^-ATPase	122,669	6.25	14.2	269	1.20	1.20	1.14	0.97	0.80	0.84	0.99
F2DZ45	Plastidic 2-oxoglutarate/malate transporter	64,625	9.72	5.9	36	0.87	0.88	1.10	1.00	1.29	1.03	1.13
A1E9J1	Photosystem I P700 chlorophyll a apoprotein A1	88,783	6.6	26.9	635	0.97	0.95	0.97	1.00	0.99	0.99	0.94
A1E9J0	Photosystem I P700 chlorophyll a apoprotein A2	87,544	6.63	21.5	2312	0.91	0.94	0.97	1.00	1.07	1.01	1.00
M0WRF7	Nitrate/chlorate transporter	51,739	9.71	7.3	34	0.82	1.00	1.35	1.03	1.24	0.75	1.20
M0WIH0	PDR-like ABC transporter	182,402	6.83	2.3	36	0.99	1.18	0.95	1.07	0.90	0.94	1.00
M0V458	Hexose transporter	56,654	8.79	23.1	244	1.01	1.01	1.10	1.10	1.03	0.94	0.94
F2D927	Pyrophosphate-energized vacuolar membrane proton pump	90,372	5.07	11.2	431	0.81	0.65	0.64	1.11	1.42	1.79	0.84
F2E844	Plasma membrane Ca^2+^-ATPase	110,168	5.43	5.6	61	1.07	1.24	1.25	1.13	0.98	0.84	1.09
M0Z2H5	Plasma membrane H^+^-ATPase-like protein	122,975	6.49	39.2	1464	0.80	0.83	0.85	1.13	1.32	1.25	0.97
Q6S5H8	Two pore calcium channel protein	94,521	5.45	4.7	52	0.98	1.07	0.97	1.13	0.93	0.94	0.88
M0WQP8	Vacuolar sugar transport	86,426	4.89	7.9	187	1.05	1.05	1.05	1.13	0.92	0.92	0.86
O48518	HvPIP1;3 protein	34,651	8.61	29.8	73	1.09	0.94	1.04	1.15	1.10	1.15	0.90
F2D2A9	Ca^2+^-ATPase	131,271	5.42	4.5	98	0.97	1.11	1.22	1.15	0.95	0.75	0.91
D2KZ38	Tonoplast intrinsic protein/Tonoplast water channel	27,332	6.04	17.6	249	0.77	0.82	0.88	1.20	1.16	1.02	0.80
M0UZZ1	Potassium transporter	97,191	8.78	3.1	32	1.04	1.26	1.07	1.32	1.00	0.98	0.95
E9LFE6	Chloride channel 1	97,124	7.88	3	54	1.25	1.38	1.24	1.43	1.05	1.06	1.02
Q08IH4	Aquaporin	34,752	8.29	20.5	88	1.16	1.10	1.22	1.61	1.24	1.18	0.85
F2D712	Apolipoprotein	66,668	9.35	15.4	124	1.27	1.45	1.40	1.64	1.05	0.96	0.93
F2DTQ3	Conserved hypothetical protein	27,453	9.79	10.4	43	1.21	1.30	1.60	1.68	1.27	0.96	0.98
F2DHE3	Reticulon-like protein	34,653	6.75	18.5	61	1.17	1.43	1.29	1.76	1.03	0.93	0.83
M0XZ33	ATPase, AAA-type, core domain containing protein	115,989	7.94	3.8	55	1.06	1.42	1.16	1.83	0.81	0.74	0.63
M0V7E0	Vacuolar ATP synthase 16 kDa proteolipid subunit	12,029	7.77	17.5	54	0.79	0.76	0.84	1.97	1.57	1.47	0.60
M0WVC5	P-glycoprotein ABCB5	169,073	9.28	3	137	1.90	1.41	2.50	2.02	1.09	0.83	0.76

The fold was the ratio by comparing relative protein abundance under salt treatment with under control conditions, or in XZ26 with in XZ169. The underlined numbers indicate significantly up-regulated (>1.5 fold) or down-regulated (<0.67 fold) proteins. UniProt ID: protein accession number in the Uniprot database (http://www.uniprot.org/). *MW* Molecular weight, *pI* Isoelectric point, *AASC* Amino acid sequence coverage, *Score* Mascot score

On the other hand, sugar transporter (M0UXN8) showed a remarkable down-regulation under high salinity in XZ169 (0.57 fold), but up-regulation for XZ26 (1.51 fold) (Table 1). However, sucrose synthase (M0UDL3) showed significant up-regulation in XZ169 under salt stress, but not in XZ26 (Table 2). Meanwhile, some proteins related to sucrose metabolism also showed up-regulation specifically in XZ169, including sucrose-phosphate synthase 2 (M0XQI1, 1.62 fold), UDP-glucose 6-dehydrogenase (M0YMY2, 1.51 fold) and glucose-6-phosphate 1-dehydrogenase (F2D6Z3, 1.57 fold). Hexokinase (M0X8J7, 1.53 fold) and pyruvate kinase (M0X6C8, 0.65 fold), two limited enzymes in glycolysis pathway, showed significant up-regulation and down-regulation in XZ169 under high salinity (Table 2), respectively, but had less change in XZ26, suggesting that glycolysis was more severely affected by salt stress in the sensitive genotype than in the tolerant genotype. The current results are consistent with those obtained in the metabolomic study. Citrate synthase (M0UG18, 1.96 fold) which is involved in TCA cycle, showed a significant up-regulation in XZ169 under high salinity, but not in XZ26. Moreover, glutamate decarboxylase (M0W9D6), glutamine synthetase (F2E708), delta 1-pyrroline-5-carboxylate synthetase (P5CS) (M0VW03), aspartate aminotransferase (M0V0J0) and asparagine synthetase (Q93XP9), had more increase in XZ169 than in XZ26 under salt stress (Table 2), resulting in a dramatic enhancement of proline and other amino acids synthesis. The result is also consistent with the findings in metabolomic analysis. On the other hand, some proteins were down-regulated in XZ169, including phosphoenolpyruvate carboxylase (M0XEC5, 0.53 fold), O-acetylserine sulfhydrylase (M0Y4H6, 0.56 fold), ribose-5-phosphate isomerase precursor (F2D226, 0.64 fold), glucose-1-phosphate adenylyltransferase (C3W8L2, 0.65 fold) and 1-aminocyclopropane-1-carboxylate oxidase 1 (F2E0G1, 0.65 fold), but not in XZ26 (Table 2). In short, the current results indicate that XZ169 were much more affected by salt stress than XZ26 in shoot proteome.

**Table 2:** The fold changes of proteins related to metabolic processes in shoots of XZ26 and XZ169 under control (CK), 200 (S200) and 400 (S400) mM salt conditions

Uniprot ID	Protein description	MW (Da)	pI	AASC (%)	Score	XZ26		XZ169		XZ26/XZ169		
						S200/CK	S400/CK	S200/CK	S400/CK	CK	S200	S400
F2DF85	Fatty acid desaturase	54,105	8.89	9.8	84	0.75	0.51	0.79	0.47	1.11	1.06	1.22
F2D126	Transcriptional coactivator/pterin dehydratase family protein	27,247	9.07	48.6	386	0.65	0.56	0.62	0.50	0.84	0.88	0.93
M0XEC5	Phosphoenolpyruvate carboxylase	124,753	5.31	32.5	503	0.83	1.18	0.67	0.53	0.18	0.22	0.40
F2D4I4	Delta-aminolevulinic acid dehydratase	51,582	5.72	30	357	0.77	0.59	0.69	0.54	0.90	1.01	0.98
M0Y4H6	O-acetylserine sulfhydrylase	46,292	6.25	7.1	62	1.06	0.88	0.87	0.56	0.74	0.90	1.16
M0WSA4	Beta-D-xylosidase	91,459	6.98	13.4	216	0.60	0.65	0.72	0.56	0.98	0.82	1.14
F2D277	Light regulated Lir1 family protein	15,228	4.62	15.7	59	0.90	0.59	0.71	0.57	1.02	1.31	1.06
F2CXV7	Chloroplast chaperonin 10	31,589	7.77	24.1	137	0.78	0.68	0.71	0.63	1.00	1.11	1.08
M0XMD8	Carotenoid isomerase 1	75,770	7.59	3.4	34	0.83	0.65	0.76	0.63	0.94	1.03	0.98
F2D226	Ribose-5-phosphate isomerase precursor	33,044	6	47.5	446	0.89	0.76	0.73	0.64	0.88	1.06	1.04
C3W8L2	Glucose-1-phosphate adenylyltransferase	63,159	8.5	39.6	586	0.88	1.23	0.70	0.65	1.37	1.73	2.61
M0X6C8	Pyruvate kinase	65,893	5.7	11.3	228	0.87	0.69	0.83	0.65	0.83	0.88	0.90
F2E0G1	1-aminocyclopropane-1-carboxylate oxidase 1	40,813	5.31	24.1	123	0.81	0.70	0.81	0.65	1.22	1.23	1.31
K9J8J5	3-phosphoglycerate kinase	20,643	6.96	80.3	1075	0.52	0.56	0.75	1.04	1.79	1.25	0.97
M0YUE3	Beta-1,3-glucanase precursor	39,312	8.92	45.9	708	1.32	1.99	1.02	1.04	0.78	1.01	1.48
B1P1S7	Xyloglucan xyloglucosyl transferase	34,460	5.95	6	80	1.18	1.57	1.23	1.12	0.84	0.81	1.18
M0YIN5	Aspartic-type endopeptidase	50,789	5.56	3.3	54	1.27	1.62	1.04	1.14	0.86	1.04	1.22
M0XNT1	S-adenosylmethionine synthase	57,814	5.8	53.3	832	1.43	1.62	1.47	1.14	1.01	0.99	1.44
M0UYW1	NADPH- protochlorophyllide oxidoreductase	54,296	9.29	20.8	365	1.78	1.31	1.92	1.23	1.12	1.04	1.19
F2DQT1	Diphosphonucleotide phosphatase 1 precursor	78,528	6.21	37	978	1.52	1.55	1.54	1.35	1.10	1.09	1.26
M0YMY2	UDP-glucose 6-dehydrogenase	65,621	5.69	16.3	156	1.38	1.16	1.51	1.47	1.14	1.04	0.89
F2CVE6	Purple acid phosphatase	81,078	6.12	3.2	35	1.25	1.56	1.37	1.51	0.91	0.84	0.94
F2E0P3	Glyoxalase/dioxygenase domain containing protein	15,948	5.45	19.7	59	1.45	1.72	1.28	1.53	0.91	1.03	1.03
M0X8J7	Hexokinase	59,384	5.48	4.8	73	1.05	1.00	1.09	1.53	0.87	0.83	0.57
M0UDL3	Sucrose synthase	120,362	8.56	2.8	29	1.07	1.28	1.54	1.54	0.98	0.68	0.81
M0UVG1	Arabinogalactan protein	25,763	7.16	12.7	44	1.05	1.21	1.33	1.56	1.08	0.85	0.83
F2D6Z3	Glucose-6-phosphate 1-dehydrogenase	68,743	6.27	17.3	247	1.35	1.28	1.36	1.57	1.06	1.05	0.86
M0Y4U4	3-ketoacyl-CoA synthase	70,202	9.66	11.1	142	1.75	1.19	1.81	1.57	1.15	1.11	0.87
F2D135	Lipase, GDSL domain containing protein	47,448	5.52	17.3	131	1.33	1.19	1.78	1.59	0.98	0.73	0.73
F2DI44	Glucosyltransferase	61,878	5.7	6.9	88	1.38	1.96	1.41	1.61	0.91	0.89	1.11
F2E4J5	Xylanase inhibitor protein I precursor	38,227	7.68	10.2	46	1.38	1.66	1.34	1.62	1.00	1.03	1.03
M0YDP9	Aspartic protease precursor	62,584	5.86	18.3	191	1.20	1.29	1.31	1.62	1.13	1.04	0.90
M0XQI1	Sucrose-phosphate synthase 2	138,496	5.81	4.1	98	1.19	1.18	1.30	1.62	0.95	0.87	0.69
M0V101	Cysteine proteinase inhibitor-I	20,157	5.5	18.3	291	1.25	1.22	1.17	1.63	1.15	1.22	0.86
M0Z0Z8	Cystathionine beta-synthase	27,238	9.18	19.5	158	1.49	1.78	1.21	1.64	0.85	1.04	0.92
M0VNQ7	D-arabinono-1,4-lactone oxidase domain containing protein	47,042	5.64	6.9	53	1.48	1.26	1.72	1.65	0.87	0.75	0.67
F2DR04	Phosphoethanolamine methyltransferase	68,971	5.15	12.6	127	1.50	1.52	1.72	1.65	1.05	0.91	0.97
M0UYT8	Alpha-galactosidase	52,595	5.42	35.2	405	1.28	1.67	1.20	1.66	1.13	1.21	1.14
M0XMF8	Ferredoxin I, chloroplast precursor	17,620	4.56	63.6	287	1.28	1.15	1.12	1.70	1.38	1.57	0.94
M0V0J0	Aspartate aminotransferase	54,313	6.11	43.9	372	0.78	0.99	2.41	1.70	0.32	0.10	0.19
M0X566	Phosphoethanolamine cytidylyltransferase	48,700	7.27	11.3	49	0.96	1.19	1.59	1.79	1.44	0.87	0.95
M0Y6F5	Raffinose synthase family protein	73,235	5.66	4.3	57	1.22	1.52	1.31	1.81	0.89	0.83	0.75
M0VT96	Photosystem II 10 kDa polypeptide, chloroplast precursor	6847	9.14	16	35	1.10	1.56	2.01	1.82	0.54	0.30	0.47
M0WPS8	Alcohol dehydrogenase	35,923	6.08	7.7	28	1.25	1.48	1.51	1.82	1.31	1.08	1.07
M0Z0G9	Polyketide reductase	41,186	5.41	9.5	103	1.43	1.86	1.39	1.88	0.76	0.78	0.75
M0XCI1	Arginine decarboxylase	79,510	5.89	10.3	91	1.35	1.37	1.44	1.89	1.01	0.94	0.73
F2DBE3	Catalase	64,999	6.58	51.4	890	1.25	1.96	1.54	1.90	1.33	1.08	1.38
M0UG18	Citrate synthase	63,414	8.87	18.9	197	1.15	1.29	1.21	1.96	1.01	0.96	0.67
Q96466	Sucrose:fructan 6-fructosyltransferase	75,184	5.2	7.5	135	1.78	1.57	1.45	2.02	1.05	1.29	0.82
B4ESE6	Papain-like cysteine proteinase	46,137	5.28	8.4	37	1.43	1.21	1.74	2.04	1.00	0.82	0.59
F2D6B1	Dehydroascorbate reductase	30,129	5.71	71.7	776	1.71	1.63	1.58	2.05	0.87	0.94	0.69
F2CWX3	Allene oxide synthase	64,971	8.93	24.2	212	1.84	1.54	1.64	2.07	1.12	1.26	0.84
M0W9D6	Glutamate decarboxylase	62,417	5.52	26	524	0.91	1.31	1.31	2.24	0.99	0.69	0.58
M0W5N8	Lysine ketoglutarate reductase	67,824	5.46	7	45	1.09	1.38	1.40	2.29	1.14	0.88	0.68
M0XN55	Pyruvate dehydrogenase E1 alpha subunit	43,225	8.53	8.1	33	1.38	1.85	1.46	2.31	0.85	0.80	0.68
M0Y4R9	Xylem cysteine proteinase 2 precursor	36,693	5.08	8.2	48	1.49	1.27	2.00	2.34	1.05	0.78	0.57
Q93XP9	Asparagine synthetase	77,293	6.14	10.9	139	1.23	1.29	2.05	2.55	1.25	0.75	0.63
F2D2K5	Acid phosphatase	34,444	9.22	25	242	2.19	1.90	2.36	2.64	1.03	0.96	0.74
F2CUQ3	Xyloglucan endotransglycosylase XET2	38,668	6.45	11.2	132	2.00	2.08	2.14	2.67	1.13	1.05	0.88
F2E708	Glutamine synthetase	45,760	5.95	8.6	49	1.11	1.51	1.37	2.76	0.93	0.75	0.51
F2CTW0	Purine and other phosphorylases	43,722	5.9	25.5	153	1.11	1.21	1.80	3.48	1.44	0.89	0.50
M0VW03	Delta 1-pyrroline-5-carboxylate synthetase (P5CS)	94,548	6.02	25.9	330	1.38	2.11	1.64	4.65	0.93	0.78	0.42
F2ELT5	Malic enzyme	78,998	6.46	8.7	66	0.73	1.65	1.79	5.38	1.86	0.76	0.57
M0Y1R9	Lipoxygenase	120,371	5.79	43.4	2310	2.18	2.02	1.93	1.97	0.90	1.02	0.93
F2CTB8	Class III peroxidase 46	44,357	5.88	9	33	2.30	2.09	1.89	2.17	1.06	1.28	1.02
F2ECQ4	Peroxidase 1	43,325	6.2	9.4	145	1.75	1.69	1.63	2.34	0.95	1.02	0.68
F2DV26	Peroxidase P7 (TP7)	36,408	9.58	7.1	61	1.80	1.66	2.08	2.57	1.04	0.90	0.67
M0Z2D5	Salt-stress induced protein (Salt protein)	18,079	7.93	13	43	2.76	2.03	2.78	4.62	0.98	0.97	0.43
M0VPJ5	Chitinase	27,237	6.93	14.7	98	3.19	2.76	4.44	6.15	0.76	0.55	0.34

See Table 1 for more details

## Discussion

High-throughput omic techniques have been used to investigate complex molecular response underlying salt tolerance in crops [24, 26–31]. However, no related study has been done to reveal the genotypic differences of wild barleys in molecular responses to salt stress. In the present study, the ionomic, metabolomic and proteomic profiles in the shoots of two Tibetan wild barley accessions were compared under different salt levels.

Salt stress causes growth inhibition of plant roots and shoots, while shoot growth is generally more severely affected [3]. The genotypic comparison in shoot ionomic responses to salt stress showed that XZ26 maintained lower Na concentration than XZ169 (Fig. 3), being consistent with its less inhibition of the shoot growth. Obviously, the difference in shoot Na concentration between the two genotypes may be a key factor attributing to the genotypic difference in shoot growth. It is commonly recognized that the salt-tolerant barley cultivars are capable for maintaining higher K/Na ratios in comparison with the sensitive cultivars under salt stress [16, 19, 24, 32]. The same result was also obtained in our previous study that relative shoot dry weight was significantly and negatively correlated with shoot Na concentration [12]. In this study, we also compared the difference of shoot proteomes between XZ26 and XZ169 under salt stress. Re-establishing homeostasis, especially for K/Na homeostasis, is considered as a critical mechanism for achieving higher tolerance in plants under salt stress [33]. To date, proton pumps (e.g. plasma membrane-ATPase and vacuolar -ATPase and vacuolar-pyrophosphatase), which provide energy source for ion transporters across plasma membrane and tonoplast, and Na/H antiporters (e.g., NHX family) and Na or K transporters (e.g., HKT family), have been identified in various plants [3, 33, 34]. In this study, although some membrane associated proteins were up-regulated in XZ169, but two plasma membrane H^+^-ATPase (F2DC32 and M0Z2H5), potassium transporter (M0UZZ1), MRP-(M0X1H9) and PDR-(M0WIH0) like ABC transporter, nitrate/chlorate transporter (M0WRF7) and chloride channel 1 (E9LFE6) remained little change in terms of protein abundance under salt stress for both genotypes (Table 1). Thus, it may be speculated that the ionic response (e.g. Na) in shoots to salt stress is probably regulated by proton pumps and ion transporters associated with root-to-shoot translocation. On the other hand, the changed pattern of other macroelements (Ca and Mg) and microelements (e.g. Cu, Fe, Mn and Zn) under salt stress is not associated with the genotypic difference in the shoot growth.

Salt-induced osmotic stress causes shoot growth inhibition immediately when Na concentration reaches a threshold level in shoots [3]. In order to adapt to osmotic stress, plants can elevate the concentration of compatible solutes in cytoplasm [34–36]. We compared the two wild barley genotypes in the response of the compatible solutes to salt stress. Compared with XZ26, XZ169 had much more proline accumulation under salinity salt stress (Fig. 5). Similar finding was also reported in the cultivated barley. Salt sensitive cultivar Clipper accumulated 4-fold higher proline in shoots than the tolerant cultivar Sahara when they were exposed to 100 mM NaCl [24]. *∆1-Pyrroline-5-carboxylase synthase* (*P5CS*), regulating proline accumulation, was rapidly induced by salt stress in Arabidopsis [37]. Correspondingly, the results of proteomic analysis also showed that the up-regulated fold of P5CS (M0VW03) was larger in XZ169 (4.65 fold) than that in XZ26 (2.11 fold) under 400 mM salt stress. In addition, some other compatible solutes, including inositol and xylitol, had also more accumulation in XZ169 than in XZ26 (Fig. 5 and Additional file 3: Table S1). Accumulation of the straight-chain polyols, mannitol, inositol and sorbitol, is reported to be correlated with stress tolerance in plants [38]. The current results indicated that XZ169 suffered from more serious osmotic stress due to higher shoot Na accumulation under salt stress compared with XZ26, and correspondingly lead to a higher accumulation of compatible solutes.

Proteomic analysis also showed that the proteins involved in ROS scavenging and defense were up-regulated in both genotypes under salt stress, including glutathione-S-transferases (F2D5L3, F2CWL1, M0YJ76 and M0XCS4), class III peroxidase (F2CTB8) and peroxidase (F2ECQ4 and F2DV26) (Table 2). Those proteins were also identified in the cultivated barley exposed to salt stress [26, 28]. Therefore it may be suggested that ROS scavenging and defense is a common mechanism of salt tolerance in barley. However, the salt-sensitive genotype XZ169 enriched more proteins to participate in those metabolic process or catalytic activities than the salt-tolerant genotype XZ26 under moderate and high salinity conditions (Additional file 4: Figure S3).

As expected, glucose concentration in shoots was dramatically reduced for both XZ26 and XZ169 under high salinity, with XZ169 having more reduction than XZ26. Widodo et al. [24] found the similar result in the shoots of two barley cultivars differing in salt tolerance exposed to long term salt stress, and no significant difference was detected between the two cultivars in glucose synthesis. On the other hand, the proteins associated with photosynthesis, including RuBisCO subunit (M0WIT3) and chloroplast chaperonin 10 (F2CXV7) were significantly down-regulated under salt stress in XZ169, but not in XZ26. In some proteomic studies, photosynthesis related proteins were also found to be down-regulated in the sensitive genotypes [26, 28]. Obviously, XZ26 was more stable in photosynthesis under salt stress than XZ169.

The two wild barley genotypes also showed the marked difference in the response of metabolisms to salt stress, with XZ26 being less affected than XZ169, on the whole (Fig. 5). The similar result was also detected in the cultivated barley [24]. Sugars act not only as osmoprotectants for maintaining osmotic balance and stabilizing macromolecules under salt stress, but also can provide energy sources to plants for growth [39]. The concentration of sucrose was not affected by salt treatment in the shoots of XZ26, but 0.47 and 0.36 fold decreases were detected in the shoots of XZ169 under moderate and high salinity, respectively (Fig. 5). Correspondingly, the protein level of sucrose synthase (M0UDL3) was significantly increased in the shoots of XZ169, but not in XZ26. Meanwhile, some proteins related to sugar metabolism were correspondingly up-regulated under salt stress in XZ169, including hexokinase (M0X8J7), sucrose-phosphate synthase 2 (M0XQI1), UDP-glucose 6-dehydrogenase (M0YMY2) and glucose-6-phosphate 1-dehydrogenase (G6PD) (F2D6Z3) (Table 2). On the other hand, up-regulated G6PD could catalyze oxidative phase in the pentose-phosphate pathway to produce more NADPH for eliminating ROS under salt stress [40], which cooperated with the enhanced glutathione-S-transferases and peroxidases as mentioned above. In short, the current results indicate that XZ26 remained less change in sugar metabolisms in comparison with XZ169 under salt stress.

It was reported that the intermediate metabolites involved in glycolysis and TCA cycle were reduced under salt stress for both barley and maize [24, 30]. In this study, the metabolites associated with glycolysis were significantly down-regulated in XZ169, being consistent with the previous findings. In contrast, the concentrations of the metabolites (e.g. citric acid and aconitic aicd) involved in TCA cycle were dramatically up-regulated under salt stress in XZ169. However, the concentrations of pyruvate and citric acid remained little change in XZ26 under salt stress relative to control (Fig. 5). On the other hand, proteomic analysis showed that the protein level of pyruvate kinase (M0X6C8) increased and protein level of citrate synthase (M0UG18) decreased in the shoots of XZ169 under salt stress, but not in XZ26. Interestingly, the response of the metabolites involved in TCA cycle to salt stress differed from that observed in the cultivated barley [24, 27]. Accordingly, the proteins involved in amino acid synthesis showed significant up-regulation in shoots of XZ169 under salt stress, leading to a dramatic increase in the concentration of amino acids (Table 2 and Fig. 5). The increased amino acid levels may not be a directly adaptive response to salt stress, but is an indicator of general stress and cell damage [20, 41]. It could be suggested that little change of metabolites in the TCA cycle is associated with a better shoot growth in the tolerant genotype (i.e. XZ26). While the sensitive genotypes (XZ169) would increase the metabolites in TCA cycle and consume more energy for its development of salt tolerance, resulting in a slower shoot growth.

## Conclusions

Compared with the sensitive genotype XZ169, XZ26 maintained a lower Na concentration in the shoots and developed superior adaptive strategies to salt stress based on the present metabolomic and proteomic studies. XZ26 had less affected proteins in metabolic processes and catalytic activities, more stable photosynthesis and less change in sugar metabolism and other energy-consuming process for better growth than XZ169. These results provide useful information for understanding molecular mechanisms of salt tolerance existed in the wild species of barley.

## Methods

### Barley materials and hydroponic culture

Seeds of Tibetan wild barley accessions XZ26 and XZ169 were disinfected for 20 min with 3 % H_2_O_2_ and rinsed several times with distilled water, then transferred onto moist filter papers in germination boxes, placing into a growth chamber (22/18 °C, day/night) in dark. After germination, light was supplied with fluorescent lamps at 250 μmol m^−2^ s^−1^. Seven-days-old seedlings were transplanted into 48-well plastic containers (35 l) with aerated hydroponic solution as described by Wu et al. [12]. Half-strength solution was used in the first 3 days and full-strength solution was supplied from the fourth day. The solution was renewed every 3 days. Seedlings were grown in a controlled growth room at 23 °C of 14 h day/18 °C of 10 h night, supplying lights with fluorescent lamps as mentioned above.

### Salt treatment and sampling

Salt treatment was initiated to the plants at the eighth day after transplanting by adding NaCl at a rate of 100 mM increment per day, to reach a final concentration of 200 and 400 mM in the hydroponics. Seedlings grew in the solution without NaCl were used as the controls.

After 7 days salt treatments, the shoots of XZ26 and XZ169 (21-days-old seedlings) from each treatment and control were sampled for determination of dry weight and ion concentration, and the shoot length was also measured. The sampled shoots were dried at 80 °C for 3 days. The relative dry weight was calculated as the ratio of each salt-treated plant to its respective control. Three biological replicates were set for both treatments and controls. For metabolomic analysis, five biological replicates of each treatment and control were sampled for metabolite extraction. For proteomic analysis, the shoots of four individual seedlings of each treatment and control were pooled as one replicate and two biological replicates were set for total protein extraction. After sampling, the shoots were frozen immediately in liquid nitrogen and stored at −80 °C for use.

### Element profiling analysis

The dried shoots were dry-ashed in a muffle furnace at 500 °C for 6 h, and then were digested as described by Wu et al. [29]. The concentrations of macroelements (Na, K, Ca and Mg) and microelements (Cu, Fe, Mn and Zn) in the digested solution were determined by an inductively coupled plasma-optical emission spectrometer (iCAP 6000 series, Thermo Fisher scientific, USA), according to the equipment operation manual. The difference of shoot element profiling among genotypes and treatments was compared using the principal component analysis (PCA) as described by Wu et al. [29].

### Metabolite profiling analysis

The shoot samples of XZ26 and XZ169 stored at −80 °C were used for metabolite extraction. Metabolites were extracted from 100 mg fresh shoot with adding ribitol as an internal quantitative standard according to Lisec et al. [42]. After extraction, the contents of metabolic compounds were determined using an Agilent 7890 gas chromatograph system coupled with a Pegasus^TM^ high-throughput time-of-flight mass spectrometer (GC-TOF/MS), according to Wu et al. [27] with some modification. Briefly, a 1 μl analyte was injected and helium was used as the carrier gas. The front inlet purge flow was 3 ml min^−1^, and the gas flow rate through the column was 1 ml min^−1^. The programs of temperature-rise was followed by initial temperature of 50 °C for 1 min, 10 °C/min rate up to 330 °C, staying at 330 °C for 5 min. The mass spectrometry data were acquired in full-scan mode with range from 85 to 600 (m/z) at a rate of 20 spectra per second after a solvent delay of 366 s.

The raw signals exacting and processing, and the normalized concentration for each metabolic compound were operated as described by Wu et al. [27]. Briefly, the data baselines filtering, peak identification and integration were imported under R software platform (http://cran.r-project.org). The TagFinder software was used for correction of retention time to mass debris, peak alignment and deconvolution analysis and the Simca-P software (version 11.5, http://www.umetrics.com/simca) was used for data normalization, employing PLS-DA model using the first principal component of VIP (variable importance in the projection) values (VIP > 1) combined with Student’s *t* test (*P* < 0.05) to find differentially expressed metabolites, and search for metabolites from commercial databases such as NIST (http://www.nist.gov/index.html) and KEGG (http://www.genome.jp/kegg). Totally, 218 metabolites were identified in the shoot metabolome of XZ26 and XZ169 under both salt treatments and control conditions. The genotypic difference of shoot metabolome was compared by employing the PCA and the PLS-DA, and the changes of metabolite were mapped to metabolic pathways according to Wu et al. [27].

### Protein profiling analysis

Shoot tissues (500 mg fresh weight) stored at −80 °C were ground using liquid nitrogen in a mortar, and transferred into 50 ml centrifuge tubes. Twenty-five milliliters of trichloroacetic acid/acetone (1:9) and 65 mM DTT were then added and precipitated for 1 h at −20 °C after thorough mixing. Mixture was centrifuged for 45 min at 8000 rpm and the supernatant was removed. Samples were air-dried and 700 μl of lysis solution (4%SDS, 150 mM Tris, pH8.0) was then added, following by ultrasonic degradation for 5 min and water bath for 30 min at 100 °C. Protein extracts were centrifuged at 14,000 g for 45 min at 25 °C. The supernatant was then collected and the concentration of proteins was determined by the Bradford kit (Bio-Rad) according to the manufacturer’s instructions.

To quantify dynamic changes of the proteome, integrated approaches composed by iTRAQ (isobaric tags for relative and absolute quantitation) labeling and mass spectrometry-based quantitative proteomics were used in the present study. The general work flows include trypsin digestion, iTRAQ labeling, HPLC fractionation, LC-MS/MS analysis, database searching and bioinformatics analysis, as described by Lan et al. [43] with some modification. Briefly, trypsin digestion of 400 μg proteins for each sample was treated with 40 μl trypsin buffer (4 μg trypsin in 40 μL dissolution buffer, Applied Biosysterms SCIEX). iTRAQ labeling were then performed using iTRAQ Reagent-8 plex Multiplex Kit (Applied Biosysterms) according to the manufacturer’s instructions. A pooled sample from all samples of the controls and treatments was set as the reference, labeling with reagent 113. The samples of XZ26 under 0, 200 and 400 mM NaCl condition were labeled with reagent 114, 116 and 117; and reagent 118, 119 and 121 for XZ169 under 0, 200 and 400 mM NaCl condition, respectively. Two independent biological experiments were conducted. HPLC fractionation was operated by Nano-HPLC EASY-nLC 1000 (Thermo Scientific). After HPLC fractionation, MS/MS analysis was performed on a Thermo Q-Exactive mass spectrometer system. The relevant parameters were as follows: the Fourier transform cell recording a window between 300 and 1800 mass-tocharge ratios (m/z); the resolution was set to 70,000 at m/z 200; and the analysis time for MS/MS was 120 min. For database searching, preliminary data were analyzed using Proteome Discoverer version 1.3 (Thermo) and MS/MS searching was performed on Mascot version 2.2 search program against NCBI (http://www.ncbi.nlm.nih.gov) database with peptide FDR ≤ 0.01.

Totally, 3358 proteins were identified in the shoots of XZ26 and XZ169 under both salt treatment and control conditions. Pearson correlation coefficient was estimated to test the repeatability of sample replicates. Highly significant positive correlation was detected between the pair-wise replicates of samples. The quantitative ratios of the identified protein over 1.5 was considered as up-regulation (*P* < 0.05), while quantitative ratio of less than 0.67 was considered as down-regulation (*P* < 0.05), when making comparison between salt treatment and control, or XZ26 and XZ169. Amino acid sequences of the differentially expressed proteins were downloaded from the UniProt-GOA database (www. http://www.ebi.ac.uk) using the protein ID and Blast-p was performed for gene ontology (GO) annotation using Blast2GO V4.0 (https://www.blast2go.com).

### Statistical analysis

The difference in the concentration of element and metabolite among treatments or between genotypes was tested by One-Way ANOVA (IBM SPSS Statistics Version 19.0) using SPSS software. The difference at *P* < 0.05 and 0.01 is considered as significant and highly significant, respectively.

## Additional files

Additional file 1: Figure S1. Relative shoot length and relative shoot dry weight of XZ26 and XZ169 after moderate (200 mM, S200) and high (400 mM, S400) salinity. (PDF 9 kb)
Additional file 2: Figure S2. The concentration of Cu, Fe, Mn and Zn in the shoots of XZ26 and XZ169 under control (CK), moderate (200 mM, S200) and high (400 mM, S400) salinity conditions. (PDF 88 kb)
Additional file 3: Table S1. The fold changes of metabolites in the shoots of XZ26 and XZ169 under control (CK), 200 (S200) and 400 (S400) mM salinity conditions. (XLSX 45 kb)
Additional file 4: Figure S3. Gene Ontology (GO) classification of the differentially accumulated proteins in the shoots of XZ26 and XZ169 after moderate (200 mM, S200) and high (400 mM, S400) salt treatments. (PDF 96 kb)
Additional file 5: Table S2. Properties of differentially expressed proteins in the shoots of XZ26 and XZ169. (XLSX 81 kb)

## Abbreviations

G6PD: Glucose-6-phosphate 1-dehydrogenase.
GO: Gene ontology
iTRAQ: Isobaric tags for relative and absolute quantitation
MS: Mass spectrometer
PC1: The first principal component
PC2: The second principal component
PCA: Principal component analysis
PLS-DA: The partial least squares-discriminant analysis
TCA: Tricarboxylic acid
