# Supplementary material for: Multi-omics analysis reveals molecular mechanisms of shoot adaption to salt stress in Tibetan wild barley
Source: BMC Genomics. 2016 Nov 7;17:889. doi: 10.1186/s12864-016-3242-9 (PMC5100661; doi:10.1186/s12864-016-3242-9)
Supplement: Additional file 1: Figure S1. — Relative shoot length and relative shoot dry weight of XZ26 and XZ169 after moderate (200 mM, S200) and high (400 mM, S400) salinity. (PDF 9 kb) [file 12864_2016_3242_MOESM1_ESM.pdf]

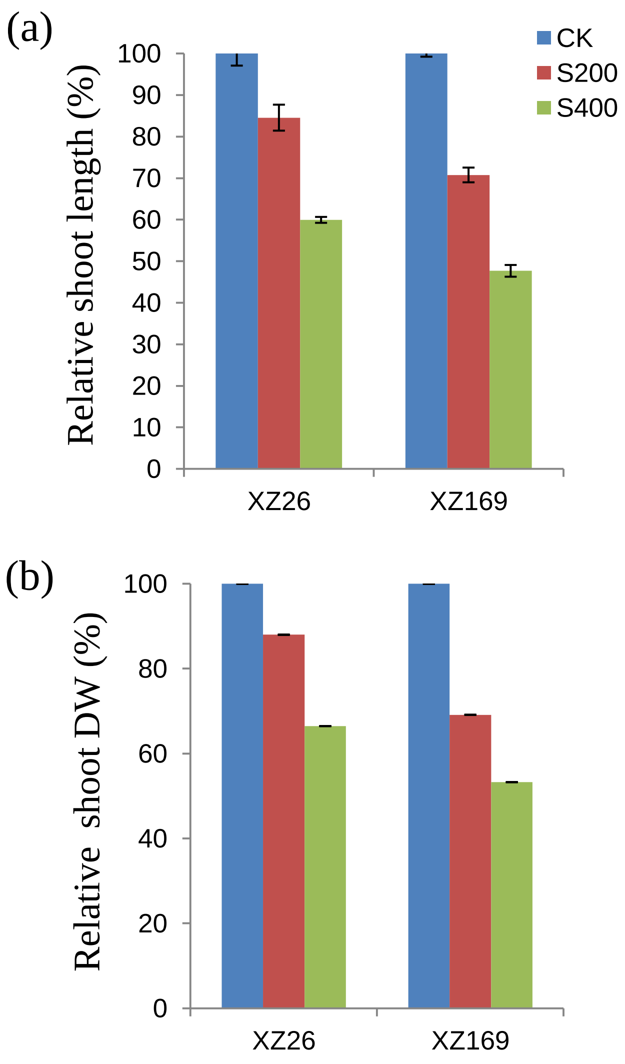

Additional files 1: Fig. 1. Relative shoot length and shoot dry weight of Tibetan wild barley genotypes (XZ26 and XZ169) after moderate (200 mM, S200) and high (400 mM, S400) salinity.
