# Supplementary material for: Multi-omics analysis reveals molecular mechanisms of shoot adaption to salt stress in Tibetan wild barley
Source: BMC Genomics. 2016 Nov 7;17:889. doi: 10.1186/s12864-016-3242-9 (PMC5100661; doi:10.1186/s12864-016-3242-9)
Supplement: Additional file 2: Figure S2. — The concentration of Cu, Fe, Mn and Zn in the shoots of XZ26 and XZ169 under control (CK), moderate (200 mM, S200) and high (400 mM, S400) salinity conditions. (PDF 88 kb) [file 12864_2016_3242_MOESM2_ESM.pdf]

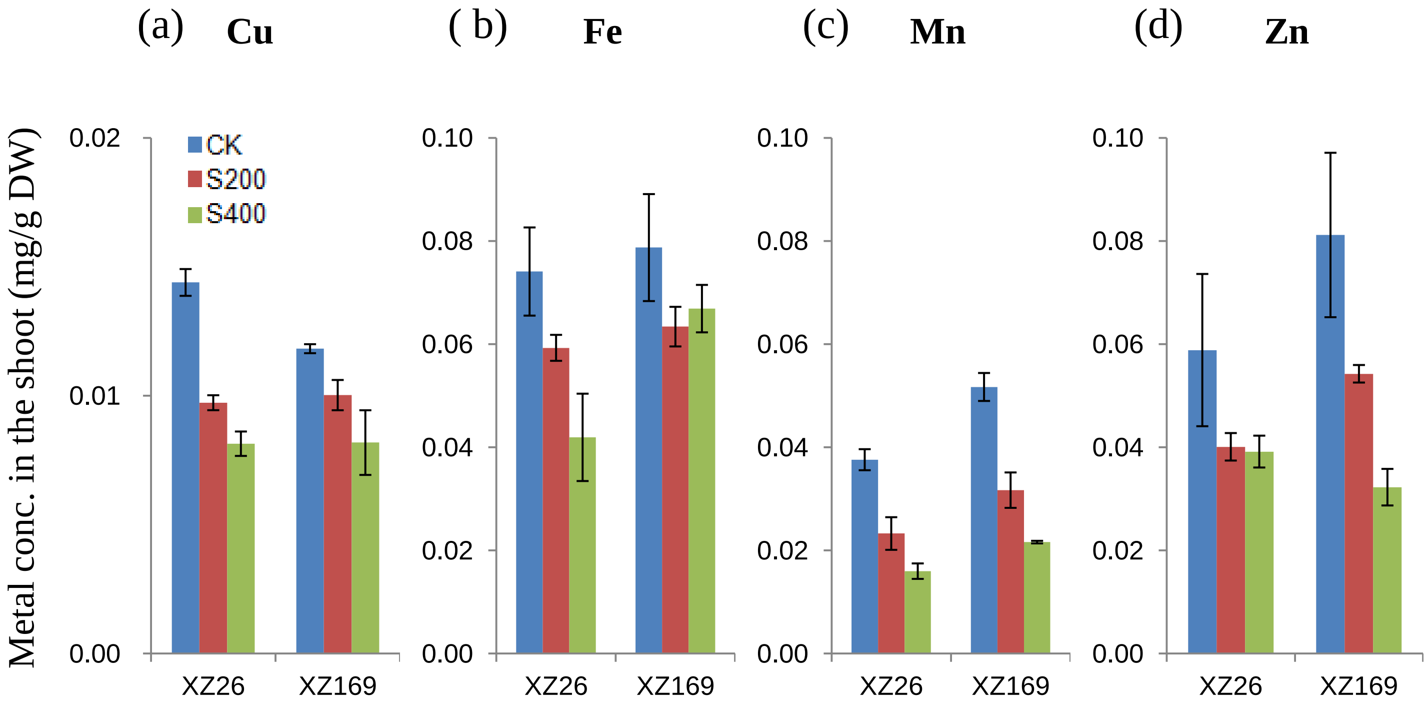

Additional files 2: Fig. 2. The concentration of Cu, Fe, Mn and Zn in the shoots of Tibetan wild barley genotypes (XZ26 and XZ169) under control (CK), moderate (200 mM, S200) and high (400 mM, S400) salinity conditions.
