# Supplementary material for: Multi-omics analysis reveals molecular mechanisms of shoot adaption to salt stress in Tibetan wild barley
Source: BMC Genomics. 2016 Nov 7;17:889. doi: 10.1186/s12864-016-3242-9 (PMC5100661; doi:10.1186/s12864-016-3242-9)
Supplement: Additional file 4: Figure S3. — Gene Ontology (GO) classification of the differentially accumulated proteins in the shoots of XZ26 and XZ169 after moderate (200 mM, S200) and high (400 mM, S400) salt treatments. (PDF 96 kb) [file 12864_2016_3242_MOESM4_ESM.pdf]

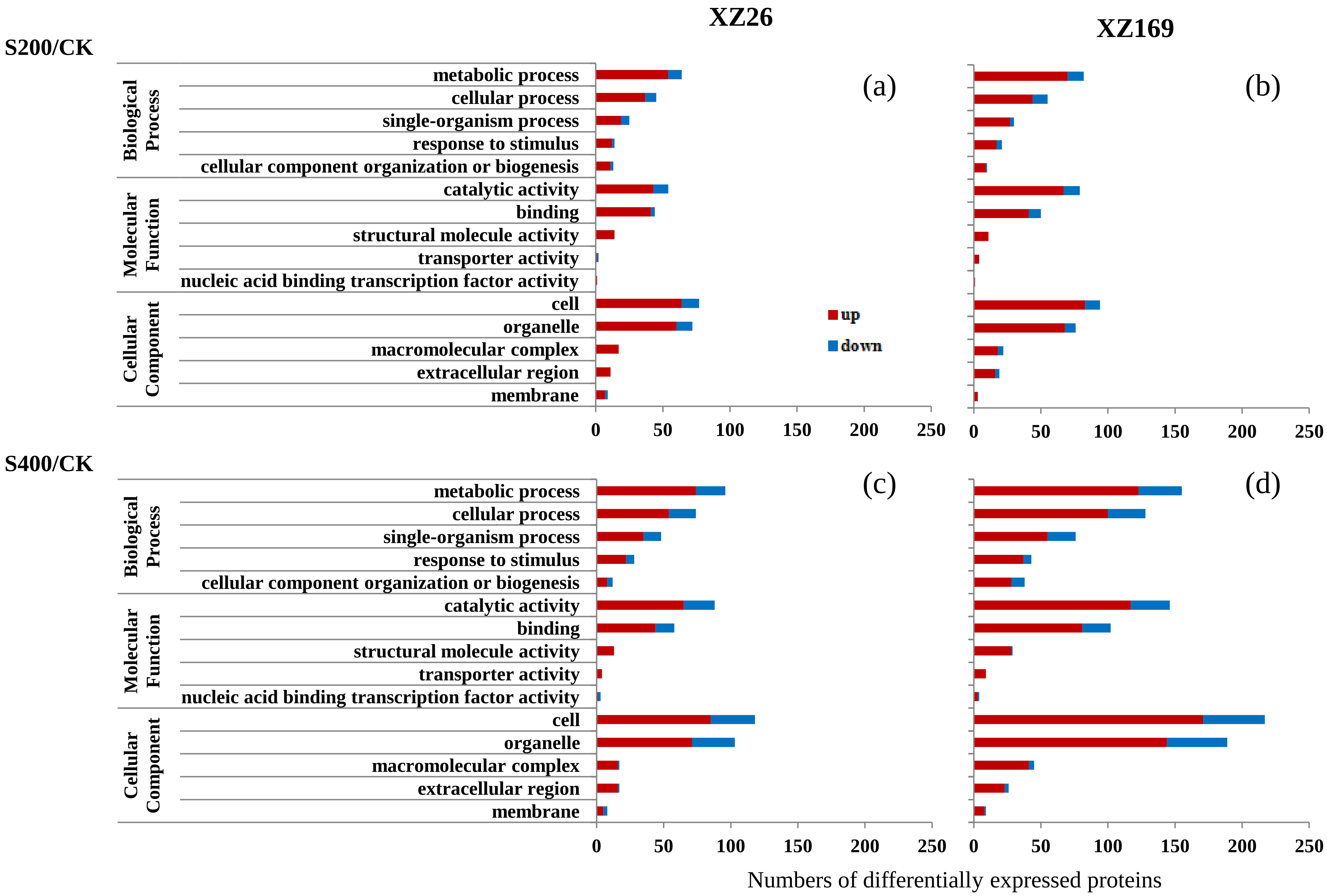

Additional files 3: Fig. 3. Gene Ontology (GO) classification of the differentially accumulated proteins in the shoots of XZ26 and XZ169 after moderate (S200) and high (S400) salt treatments.
